# Computer-Aided Diagnosis of Malignant Melanoma Using Gabor-Based Entropic Features and Multilevel Neural Networks

**DOI:** 10.3390/diagnostics10100822

**Published:** 2020-10-14

**Authors:** Samy Bakheet, Ayoub Al-Hamadi

**Affiliations:** 1 Department of Information Technology, Faculty of Computers and Information, Sohag University, Sohag P.O. Box 82524, Egypt; 2 Institute for Information Technology and Communications (IIKT), Otto von Guericke University Magdeburg, 39106 Magdeburg, Germany; ayoub.al-hamadi@ovgu.de

**Keywords:** computer-aided diagnosis, dermoscopy, skin cancer, melanoma skin cancer, Gabor-based entropic features, level neural network, cross-validation

## Abstract

The American Cancer Society has recently stated that malignant melanoma is the most serious type of skin cancer, and it is almost 100% curable, if it is detected and treated early. In this paper, we present a fully automated neural framework for real-time melanoma detection, where a low-dimensional, computationally inexpensive but highly discriminative descriptor for skin lesions is derived from local patterns of Gabor-based entropic features. The input skin image is first preprocessed by filtering and histogram equalization to reduce noise and enhance image quality. An automatic thresholding by the optimized formula of Otsu’s method is used for segmenting out lesion regions from the surrounding healthy skin regions. Then, an extensive set of optimized Gabor-based features is computed to characterize segmented skin lesions. Finally, the normalized features are fed into a trained Multilevel Neural Network to classify each pigmented skin lesion in a given dermoscopic image as benign or melanoma. The proposed detection methodology is successfully tested and validated on the public PH2 benchmark dataset using 5-cross-validation, achieving 97.5%, 100% and 96.87% in terms of accuracy, sensitivity and specificity, respectively, which demonstrate competitive performance compared with several recent state-of-the-art methods.

## 1. Introduction

A recent report issued by the National Cancer Institute (NCI) stated that skin cancer is the most common cancer among people between the ages of 25 and 29 in the United States. The main types of skin cancer are squamous cell carcinoma, basal cell carcinoma, and melanoma. Although melanoma is much less common than the other types of skin cancer, it is much more likely to invade nearby tissue and spread to other parts of the body. In other words, melanoma accounts for only about 1% of skin cancers, but it causes a large majority of skin cancer deaths [1]. Moreover, melanoma is most frequently diagnosed among people aged 65–74 and the greatest percentage of melanoma deaths occur among people aged 75–84. An estimated 100,350 new cases of melanomas and 6850 deaths from the disease are expected to occur in the United States in 2020. From a clinical point of view, melanoma is a skin cancer type that harms DNA (mutations) in skin cells, causing uncontrolled growth of these cells. It develops from the melanocyte, a melanin-producing cell located in the stratum basale of the epidermis. Clinical evidence that melanoma typically occurs in the skin, but may rarely occur in the eye, intestines, or mouth. The major known exogenous risk factor for melanoma is excessive exposure to ultraviolet (UV) radiation. Meantime, a personal history of sunburn, giant congenital nevi, genetic mutations, and a case history of melanoma all increase the risk of developing melanoma. A crucial method to assist within the diagnosis of melanotic lesions is Epiluminescence Microscopy (ELM), also known as dermatoscopy [2] that allows for the magnification of lesions, while simultaneously providing a polarized light source rendering the stratum cornea translucent. For experienced users, dermoscopy is generally believed to be more accurate than clinical examination for the diagnosis of melanoma in pigmented skin lesions. The diagnostic accuracy of dermoscopy is likely to be mostly dependent on dermatology training.

An automated computer-aided diagnosis (CAD) system for diagnostic melanoma typically goes through three basic steps or phases: (i) image preprocessing and skin lesion segmentation, (ii) extraction and selection of the lesion features, and (iii) classification of the skin lesions. Fundamentally, the first step involves preprocessing of the image data, such as image resizing, color space conversion, contrast enhancement, noise reduction and hair removal. In the second step, segmentation of skin lesions (i.e., regions of interest (ROIs)) is performed in order to separate pigmented skin lesions from the healthy surrounding skin. During the feature extraction process, each skin lesion is processed and a set of specific dermoscopic characteristics (i.e., visual descriptors) similar to those visually recognized by expert dermatologists, such as color, asymmetry, border irregularity, differential structures, is determined and computed from the segmented skin lesion to accurately describe a melanoma lesion. Finally, the extracted features from the skin lesion are fed to the feature classification module to classify each skin lesion into either benign or malignant class. The remainder of the paper is organized as follows. Section 2 presents a summary of the related work. The proposed detection method is described in Section 3. Section 4 is devoted to the experimental results and performance evaluations. Finally, in Section 5, conclusions are drawn and some perspectives for future work are given.

## 2. Related Work

In the past two decades or so, epidemiological data have revealed that a dramatic increase in the incidence and mortality of melanoma skin cancer could be observed worldwide. Therefore, many researchers in the fields of computer vision and medical image understanding have long been interested in developing high performance automatic techniques for skin cancer detection from dermoscopic images [3,4,5,6,7]. Thanks to the efforts of such researchers, several clinical decision rules (CDRs) devised by dermatologists were established in an attempt to identify partial skin lesions. Some of these algorithmic methodologies effectively incorporated in diagnosing pigmented lesions from dermoscopic images include classical pattern analysis [8], ABCD rule [9], Menzies method [10], and seven-point checklist [11].

Automatic skin lesion segmentation is a crucial prerequisite yet challenging task for CAD of skin cancers. The segmentations challenge can be attributed to an interplay of a range of factors, such as illumination variations, irregular structural patterns, the presence of hairs, as well as the existence of multiple lesions in the skin [12,13,14,15]. As mentioned earlier, several different methods and algorithms have been developed to automatically segment skin lesion images, including histogram thresholding [16,17], clustering [18,19], active contours [20,21], edge detection [22,23], graph theory [24], and probabilistic modeling [25]. Feature extraction to describe skin lesions is considered as the most crucial task in the automatic classification and diagnosis of skin lesions.

The most common approach to identifying the physical characteristics of melanoma is a rule mentioned as ABCD skin cancer [26]. In [27], an automated system for melanoma detection is proposed using Support Vector Machines (SVMs) and a set of discriminating features extracted from the intrinsic physical attributes of skin lesions such as asymmetry, border irregularity, color variation, diameter, and texture of the lesion. Another most related work is presented in [28], where a real-time framework for melanoma detection is proposed using an SVM classifier and a set of optimized HOG-based features. Moreover, in [29], two hybrid techniques based on feed forward backpropagation artificial neural networks and k-nearest neighbors are proposed for skin melanoma classification. The obtained results have shown that the proposed techniques are robust and effective.

## 3. Proposed Methodology

**Figure 1:**
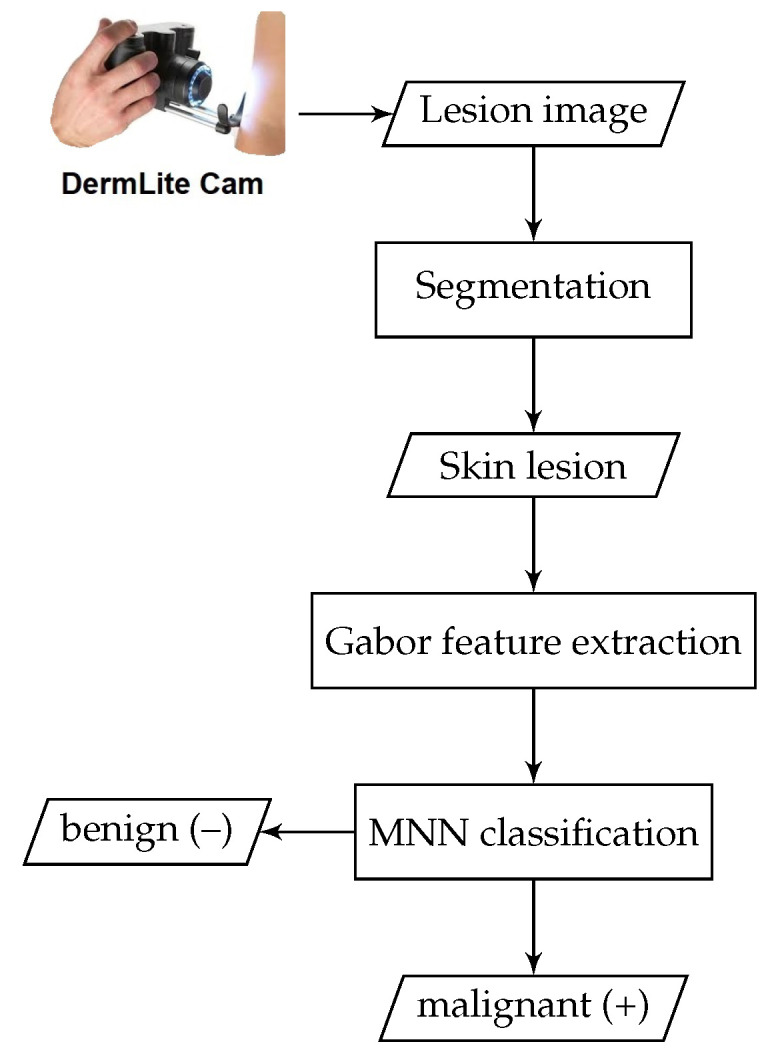
Block diagram of the proposed CAD system for melanoma detection.

In this section, the proposed methodology for automatically detecting skin cancer is described. A brief conceptual block diagram depicting the details of the proposed system operation is given in Figure 1. The general structure of the proposed framework works as follows: As an initial step, the skin lesion region that is suspected of being a melanoma lesion is segmented from the surrounding healthy skin regions, by applying iterative automatic thresholding and morphological operations. Then, an optimized set of local Gabor-based texture features is extracted from the skin lesion region. A one-dimensional vector representation is generated from the extracted Gabor features and then fed into a neural model for skin lesion classification. The details of each part of the proposed method are described in the following subsections.

### 3.1. Image Preprocessing

The image preprocessing step is basically responsible for detecting and reducing the amount of artifacts from the image. In dermoscopy images, this step is necessary, since many dermoscopy images include a lot of artifacts such as skin lines, air bubbles and hair that have to be removed to diagnose skin cancer correctly. Incorrect segmentation of pigmented lesion regions can occur, if such artifacts are not removed or inhibited. Here, the preprocessing involves three main processes: (i) image resizing and grayscale conversion, (ii) noise removal by applying a simple 2D smoothing filter, (iii) image enhancement.

### 3.2. Skin Lesion Segmentation

Skin lesion segmentation is a part of computer-aided skin cancer detection. Automated skin lesion segmentation is the most crucial step toward the implementation of any computer-aided detection system for skin cancer. For the segmentation of skin lesions in an input dermoscopy image, the presented method involves iterative automatic thresholding and masking operations, which are applied to the enhanced input skin lesion images. The segmentation procedure begins with applying automatic thresholding proposed by the Otsu method [30] for each of the R, G and B planes in the input image. Binary masks for each plane are then obtained and combined to create a final lesion mask. We use a 3-plane masking procedure in order to increase segmentation accuracy.

The segmented image may contain some smaller blobs that are actually not skin lesions. To overcome this problem, a common solution is to employ morphological-area opening on the segmented image. Finally, a finer segmented image that contains only the skin lesions can be obtained by smoothing the binary image using a series of gradually decreasing filter sizes using an iterative median filter technique (i.e., 7×7,5×5 and 3×3). Additionally, in order to avoid the detection of extremely small non-skin lesions and to avoid confusion between isolated artifacts and objects of interest, we take extra precautions by applying two additional filters to ensure that they correspond to the skin lesions of interest. First, an adaptive morphological open-close filter is iteratively applied to the resulting binary image to remove objects that are too small from the binary image, while maintaining large objects in shape and size. This filter is ideally carried out using a cascade of erosion and dilation operations using locally adaptive structuring elements.

**Figure 2:**
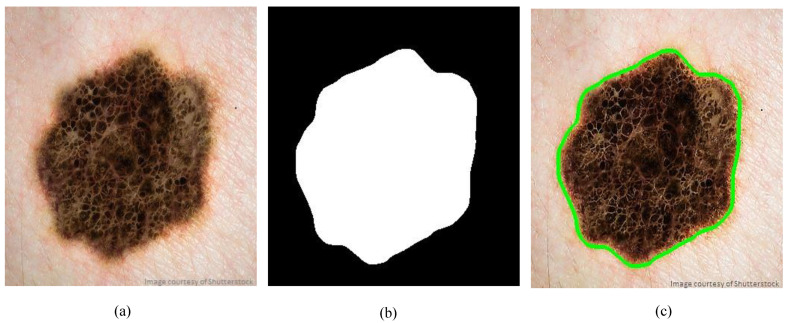
Sample segmentation of skin lesions: (**a**) Original dermoscopy image, (**b**) Binary mask, (**c**) Traced skin lesion

Furthermore, the so-called size filter is applied as a second filter to remove objects of size less than a specified threshold. Once the size filter is applied, almost all spurious artifacts of less than 5% of the image size will be erased from the binary image. However, all contours are detected by applying a modified canny edge detector [31] after filtering out of all irrelevant image elements and isolated objects. The segmentation results shown in Figure 2 demonstrate that the proposed method can correctly and precisely segment the skin lesion from the surrounding normal skin tissues.

### 3.3. Gabor Feature Extraction

Gabor wavelets are widely used to extract texture information from images at different frequencies and orientations [32]. In this subsection, we show how to extract interpretative features for skin lesion discrimination and how to derive a new texture descriptor, a so-called Gabor–Fisher descriptor (GFD), which is invariant to scale, rotation and changes in illumination.

#### 3.3.1. 2D Gabor Filters

Due to their unique distinctive properties, texture features based on Gabor wavelets have been dominantly applied in many diverse application fields, such as pattern recognition, data clustering and signal processing. Gabor kernels exhibit highly desirable characteristics of capturing spatial locality and orientation selectivity and are optimally localized in the space and frequency domains [33,34]. Hence, they have the capacity to extract highly discriminative features to describe target objects in a given image. A 2D Gabor filter is typically formulated as a Gaussian modulated sinusoid in the spatial domain and as a shifted Gaussian in the frequency domain. The Gabor wavelet [35] representation of an image allows description of spatial frequency structure in the image, while maintaining information about spatial relations. A family of Gabor wavelets (kernels, or filters) is formally expressed as a product of an elliptical Gaussian envelope and a complex plane wave, as follows

(1) $$\psi_{j}\left(z\right)=\frac{|k_{j}{|}^{2}}{\sigma^{2}}e^{-\frac{|k_{j}{|}^{2}{\left|z\right|}^{2}}{2\sigma^{2}}}\left[e^{-ik_{j}z}-e^{-\frac{\sigma^{2}}{2}}\right]$$

where $z=x+iy,i=\sqrt{-1}$ and |·| denotes the norm operator. The wave vector $k_{j}$ is defined as follows,

(2) $$k_{j}=k_{v}e^{-i\phi_{\mu }},k_{v}=2^{-\frac{v+2}{2}}\pi ,\phi_{\mu }=\mu \frac{\pi }{8}$$

**Figure 3:**
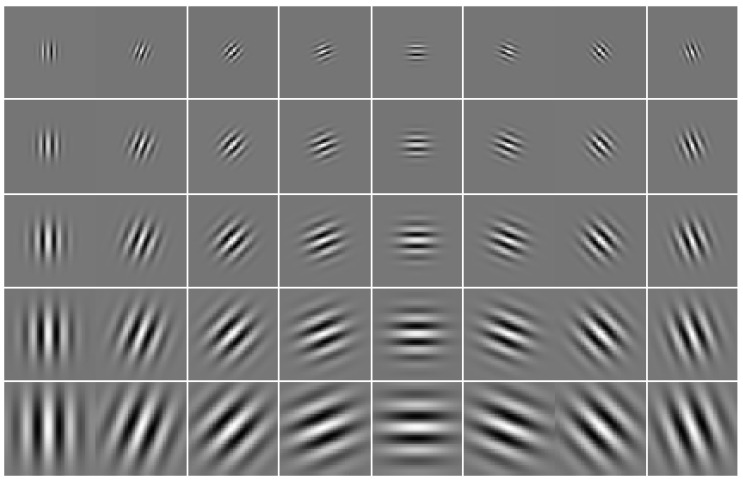
Real part of 40 Gabor kernels at 5 scales and 8 orientations.

The index j=μ+8v, where μ and *v* denote the orientation and scale of Gabor kernels, respectively. Figure 3 shows 2D plots of the real part of a set of Gabor kernels with 40 coefficients (5 spatial frequencies and 8 orientations).

#### 3.3.2. Extracted Local Gabor Features

The set of complex coefficients for Gabor kernels of different frequencies and orientations at a pixel is called a *jet*. A jet that holds the responses of Gabor convolutions at each pixel *z* in a given image *I* can be defined based on a wavelet transform, as follows,

(3) $$J_{j}\left(z\right)=\int I\left(\overset{´}{z}\right)\psi_{j}\left(z-\overset{´}{z}\right)d^{2}\overset{´}{z}$$

Once a series of proper Gabor filters (i.e., kernels of Gaussian functions modulated by sinusoidal plane waves) with different frequencies and orientations are determined and applied at different locations of the image, Gabor features can be yielded by simply convolving the image with these kernels, as given in Equation (5). In the presented work, we initially adopt a filter bank comprised of 40 log-Gabor filters (5 scales and 8 orientations) to extract local texture features from skin lesions (i.e., ROIs) in a given dermoscopy image.

(4) $$\begin{array}{ccc} k_{v} & = & 2^{-\frac{v+2}{2}}\pi ,\;v=0,\ldots ,4\\ \phi_{u} & = & \frac{\pi }{8}u,\;u=0,\ldots ,7 \end{array}$$

More formally, the resultant Gabor features at (x,y) location include the output of the convolution of a bank of all 40 Gabor filters with a pixel at (x,y) in the skin lesion (more precisely, ROI):

(5) $$s=\{\Psi_{u,v}\left(x,y\right):u\in \left\{0,\ldots ,7\right\},v\in \left\{0,\ldots ,4\right\}\}$$

**Figure 4:**
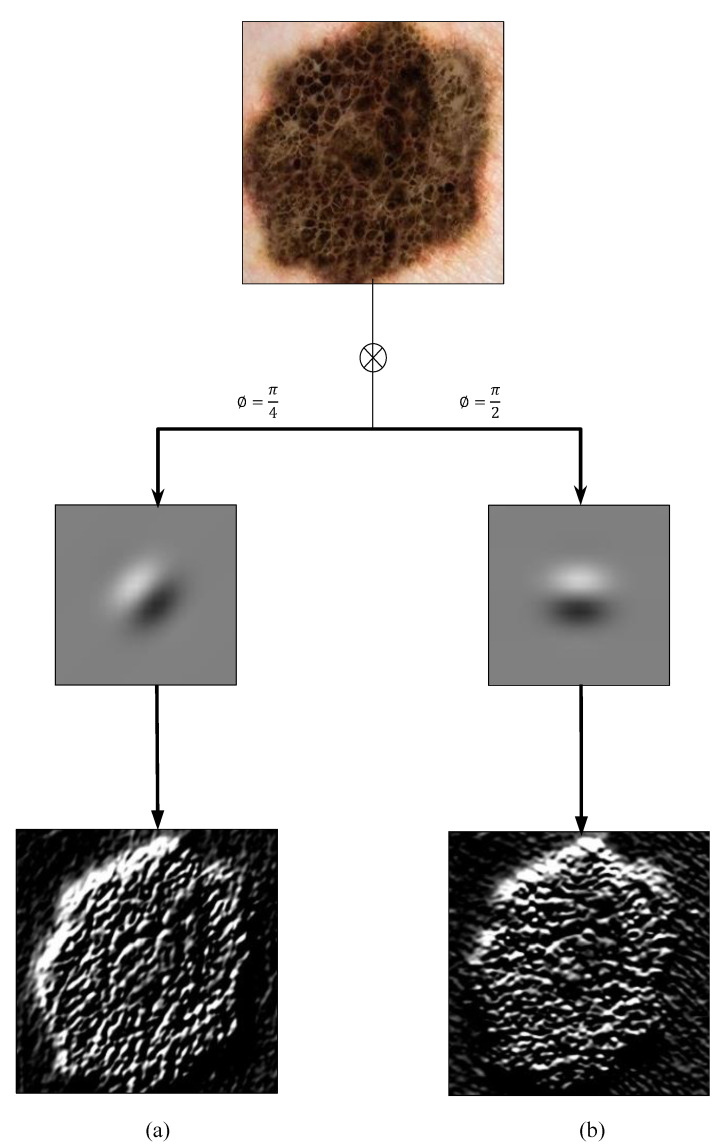
Gabor filter responses of a sample skin lesion at orientation angles of (**a**) $\frac{\pi }{4}$ and (**b**) $\frac{\pi }{2}$, respectively.

Figure 4 shows the convolution results from the application of two Gabor filters on a sample skin lesion at orientation angles of $\frac{\pi }{4}$ and $\frac{\pi }{2}$, respectively. Strictly speaking, for a patch dermoscopy image of size M×N, the final convolution of a patch dermoscopy image with a bank of 40 Gabor filters will result in a feature vector of 5×8×M×N=40M×N.

Due to the fact that the parameters of Gabor filters are selected experimentally, it is likely that the computed features contain a large number of irrelevant/redundant information (e.g., highly correlated features), which can seriously degrade the performance of learning models in terms of accuracy and computational time. To reduce the interference of redundant information contained in lesion features, an efficient feature selection technique should be performed to de-correlate the extracted features and to drastically reduce their dimensionality, while retaining a good learning performance.

In many object recognition and classification applications, the image-mean, image-standard deviation, and/or image-energy are routinely employed to select the most useful features. Instead, in the current work, we opt to follow a different approach that turns out to be a more effective strategy for achieving the goals of feature reduction and selection of most relevant features. To this end, the Gabor filter outputs are initially normalized to strengthen the convolved images having spatially distributed maxima. Then, the so-called nonextensive entropies (e.g., Rènyi entropy and Tsallis entropy) and Fisher information (FI) are calculated from the normalized Gabor filter magnitude responses as follows,

(6) $$\begin{array}{ccc} H_{1}\left(P\right) & = & \frac{1}{1-\alpha }\mathrm{lg}\left[\sum_{i}p_{i}^{\alpha }\right],\;\alpha \geq 0,\alpha \neq 1\\ H_{2}\left(P\right) & = & \frac{1}{\alpha -1}\left[1-\sum_{i}p_{i}^{\alpha }\right],\;\alpha \geq 0,\alpha \neq 1\\ F(P) & = & \sum_{i}\frac{{\left(p_{i+1}-p_{i}\right)}^{2}}{p_{i}} \end{array}$$

where *P* is a probability distribution estimation obtained from the histograms of Gabor filter responses, $H_{1}$ and $H_{2}$ are the Rènyi and Tsallis formalisms [36] of generalized nonextensive entropies, respectively, and *F* is the Fisher information measure. At this point it is worth mentioning that the primary motivation for considering this effective feature selection scheme is not only to reduce computational complexity of feature extraction, but also to guarantee a reasonably good learning performance. Due to their robustness with respect to occlusion and geometrical transformations, there is widespread agreement that local features provide much more stability than global features in most operational applications, therefore they are generally perceived to be the most effective tool for object representation and detection tasks [37,38].

**Figure 5:**
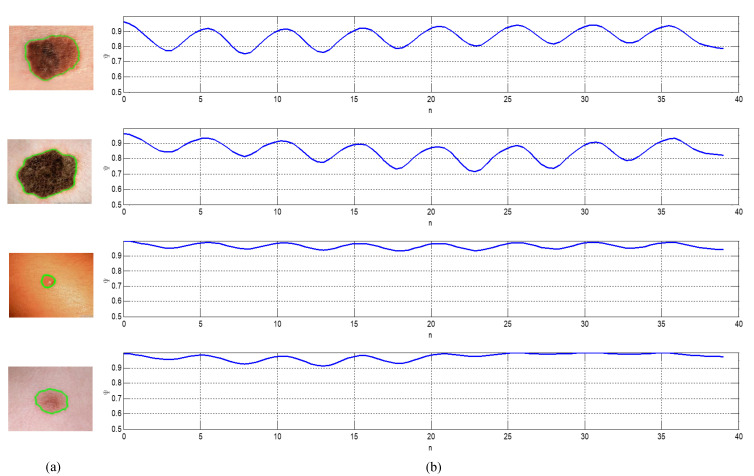
A sample of four skin lesion images and the plots of its local Gabor descriptors: (**a**) Original lesion image, (**b**) local Gabor descriptors; the first two dermoscopy images are malignant melanoma cases, while the other two images are benign lesions (from top to bottom, respectively).

Figure 5 shows a sample of four skin lesion images along with the corresponding plots of their local Gabor-based feature descriptors. The first two dermoscopy images are malignant melanoma cases, while the other two images are benign lesions (from top to bottom, respectively). At this point, it is worthy to emphasize that before concatenation, each attribute of the local features is normalized into [0,1] to allow for equal weighting among each type of feature. The normalized features are then fed to the evolved MNN for feature classification. Additionally, we would like to argue that the normalized Gabor feature descriptors provide the potential for more accurate and reliable feature extraction, that in turn has a significantly positive impact on the performance of the proposed CAD system for melanoma detection.

### 3.4. Skin Lesion Classification

The goal of this section is to describe the classification module, which we employ in the proposed MNN architecture for diagnosing melanoma lesions. Generally, the main purpose of the classification module in the proposed framework is to discern the Gabor-based features extracted from skin lesions in order to classify the skin lesions in dermoscopic images into melanoma or benign nevus. The accuracy and robustness of the classification module with a supervised learning strategy are primarily based on the availability of sufficient labeled dermoscopic images (i.e., training set). Hence, the learning strategy in this case, is simply referred to as supervised learning. In the existing literature, there are several classification techniques that are exactly tuned to be reliably applicable for classifying skin lesions in dermoscopy images, such as Artificial Neural Networks (ANNs), Support Vector Machines (SVMs), Naïve Bayesian (NB), k-Nearest Neighbor (k-NN), and Conditional Random Fields (CRFs) [39,40,41,42,43].

In the current work, the detection task of skin lesions is formulated as a typical binary classification problem, where there are two classes for skin lesions and our goal is to assign each skin lesion in given dermoscopy images an appropriate diagnostic label (i.e., malignant melanoma or benign nevus). There are plenty of existing supervised learning algorithms [44,45,46] that can potentially train an effective detector for skin malignancy. Due to its good reputation as a highly accurate paradigm and its excellent generalization capability, in the current diagnostic framework, we propose to employ an evolved neural model (so-called Multilevel Neural Network) for the classification task.

The neural classification model offers several generic advantages over other competitive machine learning. (ML) models, including, for example, the easiness of training, high selectivity, high rapidity, realistic generalization capability and potential capability to create arbitrary partitions of feature space. In its standard form, the traditional neural model, however, is limited by its low classification accuracy and poor generalization properties, due to the dependence of its neural units on a standard bi-level activation function that permanently produces a binary response. To cope with this restriction and allow the neural units to produce multiple responses, a new functional extension for the standard sigmoidal functions should be created [44]. This functional extension is termed Multilevel Activation Function (MAF), and hence the neural model employing this extension is termed as Multilevel Neural Network (MNN).

**Figure 6:**
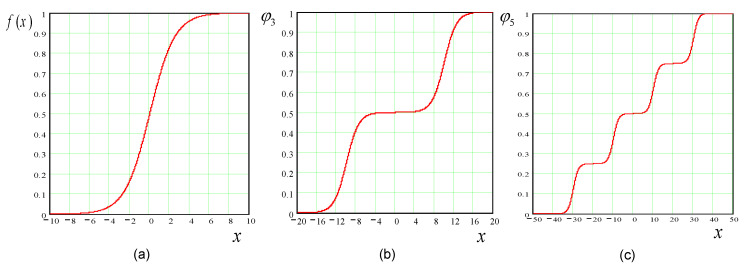
Standard sigmoidal function and its multilevel versions: (**a**) Sigmoidal function; (**b**) Multi-level function for r=3; (**c**) Multi-level function for r=5.

There are various multilevel versions corresponding to several standard activation functions. A multilevel version of an activation function can be straightforwardly derived from its bi-level standard form as follows. Assuming the general form of a standard sigmoidal function f(x) depicted in Figure 6a is defined as:

(7) $$f\left(x\right)=\frac{1}{1+e^{-\beta x}}$$

where β>0 is an arbitrary constant, i.e., the steepness parameter. Therefore, the multilevel versions of the activation function can be directly derived from Equation (7) as follows,

(8) $$\varphi_{r}\left(x\right)\leftarrow f\left(x\right)+\left(\lambda -1\right)f\left(c\right)$$

where λ is an index running from 1 to r−1, *r* is the number of levels, and *c* is an arbitrary constant. Multilevel sigmoidal functions for r=3 and 5 are depicted in Figure 6b,c, respectively.

**Figure 7:**
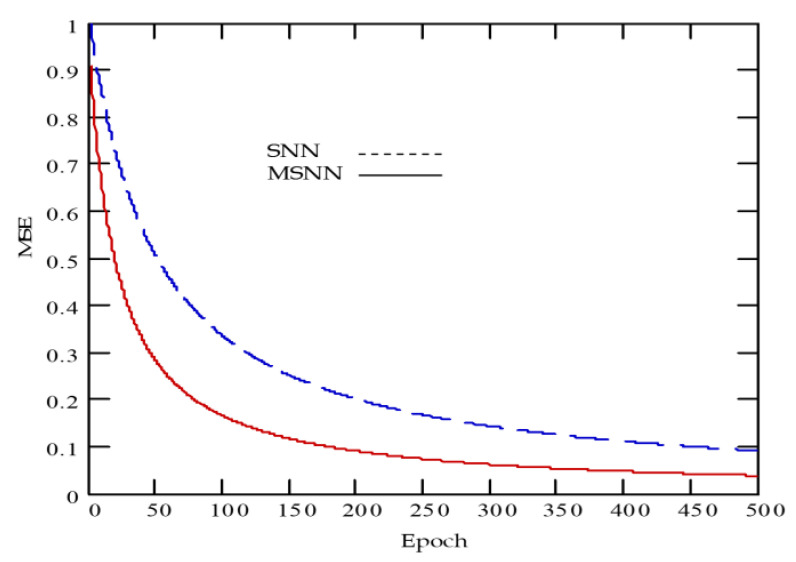
Averaged learning curve comparison between sigmoidal neural network (SNN) and multilevel sigmoidal neural network (MSNN) models.

**Figure 8:**
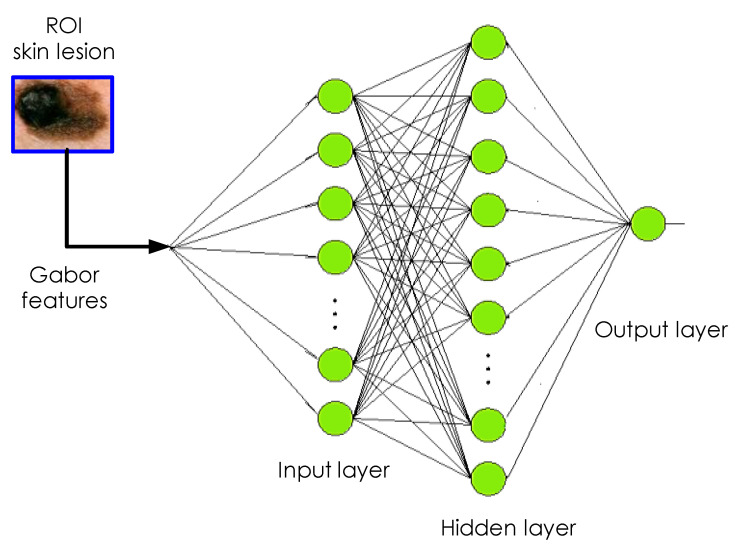
The MNN structure established for melanoma detection.

At this point, it is worthwhile mentioning that it was experimentally reported that the neural classifier employing multilevel functions is able to maintain a superior learning performance over its neural counterpart employing traditional sigmoidal functions [44], as depicted in Figure 7. The evolved MNN model is an effective diagnostic approach that is normally made up of three layers, namely input layer, hidden layer and output layer, for which two nearby layers are fully-connected [47], see Figure 8.

In the proposed framework, the model parameters are learned via a second-order local algorithm very similar to the well-known Levenberg–Marquardt (LM) algorithm [48]. Such an algorithm is fast and most appropriate for training simpler structures under the Multilayer Perceptron (MLP) architecture. Furthermore, the algorithm, which is a special combination of the error backpropagation and Gauss–Newton algorithms, makes use of a conjugate gradient method by introducing the local Jacobian matrix instead of the Hessian matrix. In the beginning of training, the weights are initialized randomly. There will be known inputs and a desired output. The MNN model provides the actual output that is compared to the desired output during each epoch. An error is produced when both outputs are not equal. This error is propagated backward and weights are updated such that the training is halted, when the difference between the desired output and the actual output is minimized.

## 4. Experimental Results

**Figure 9:**
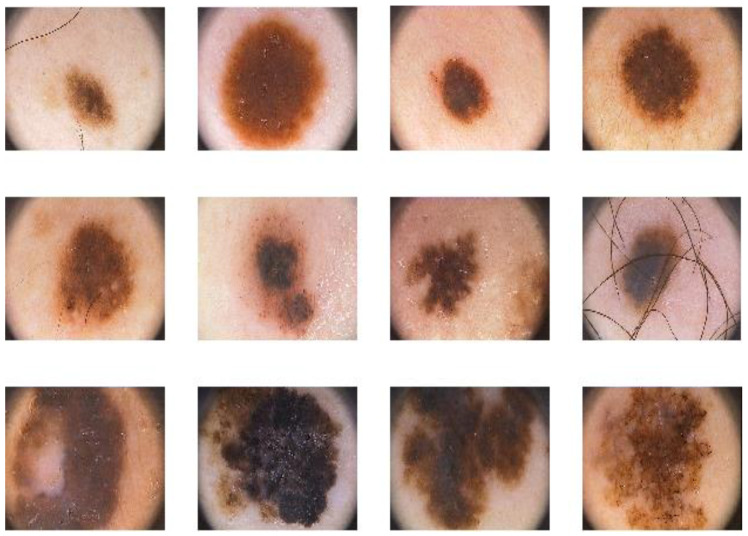
A sample of images from the PH2 dermoscopic dataset: common nevi (row 1), atypical nevi (row 2) and melanomas (row 3).

In this section, the experimental results obtained are shown and discussed in order to demonstrate the performance of the proposed malignant melanoma detector. The performance of the proposed system is tested on the PH2 benchmark dataset [49] that consists of a total number of 200 8-bit RGB dermoscopic images of melanocytic lesions with a resolution of 768×560 pixels. The images include different types of skin lesions: 80 common nevi, 80 atypical nevi, and 40 melanomas. The dermoscopic images were created at the Dermatology Service of Hospital Pedro Hispano (Matosinhos, Portugal) under the same conditions through the tuebinger mole analyzer system using a magnification of 20 times. Figure 9 shows a sample of the lesion images contained in the PH2 dataset.

For computational efficiency, all images are resized to a fixed dimension of 256 × 256 pixels, as a preprocessing step prior to the feature extraction phase. The images in the dataset are then split randomly into two subsets, one as a training set (80%) and the other as a test set (20%) and the cross-validation procedure is performed in order to estimate how accurately the detection model will perform in an independent test set.

In the current neural architecture, there are two hidden layers of 10 neurons each, while the output layer has only one neuron (see Figure 8) that gives an output of 0 for non-cancerous (or benign) or 1 for cancerous (or malignant). Each neuron employs a multilevel sigmoidal activation function. The neural model is trained on the features extracted from lesion regions through backpropagation. In the backpropagation algorithm, the training process iteratively proceeds until the Mean Square Error (MSE) between the network output and desired output computed over entire epoch achieves a minimum value (i.e., less than a pre-set threshold) or the number of iterations reaches a specified value. To validate the proposed method, 5-fold cross-validation was used in our experiments. More specifically, out of total image samples, in each K fold, 160 images are chosen for training and the remaining 40 images are used for testing the performance of the trained neural model.

For performance evaluation of the proposed system, the obtained results are quantitatively assessed in terms of three commonly used performance indices, namely, sensitivity (SN), specificity (SP), and accuracy (AC). The three indices are defined as follows:

Accuracy is the probability that a randomly chosen instance (positive or negative, relevant or irrelevant) will be correct. More specifically, accuracy is the probability that the diagnostic test yields the correct determination, i.e.,

(9) $$AC=\frac{TP+TN}{TP+TN+FP+FN}\times 100\%$$

Sensitivity (also called true positive rate or recall) generally refers to the ability to identify melanoma case positively, i.e.,

(10) $$SN=\frac{TP}{TP+FN}\times 100\%$$

Specificity (also called true negative rate) refers to how well a test recognizes patients who do not have a disease, i.e.,

(11) $$SP=\frac{TN}{TN+FP}\times 100\%$$

where TP (true positive) is the correctly predicted positive cases, TN (true negative) is the correctly predicted negative cases, FP (false positive) is the incorrectly predicted negative cases, and FN (false negative) is the incorrectly predicted positive cases. Table 1 presents the cross-classification table: standard-of-reference benign/malignant vs. model’s prediction benign/malignant.

Based on the figures in Table 1, it can be calculated that the positive predictive value (PPV) and negative predictive value (NPV) of the diagnostic model are 88.9% and 100%, respectively. Furthermore, the performance of the proposed diagnostic model is appraised in terms of overall accuracy, sensitivity, and specificity. The obtained results revealed that our diagnostic framework can achieve 97.50%, 100% and 96.87% for overall accuracy, sensitivity and specificity respectively, for 5-fold cross-validation. With regard to the confidence, the proposed diagnostic approach achieved an average ROC area under the curve (AUC) of 0.94 (95% confidence interval: 0.92–0.96). Moreover, the approximated 95% confidence intervals of sensitivity, specificity, PPV, and NPV are 100%, 95–99%, 86–90% and 100%, respectively, where the confidence level was typically set to 95%.

In order to quantify the effectiveness of the proposed approach, a comparison of our framework with other standard state-of-the-art works [28,29] has been conducted and analyzed. This comparison is summarized in Table 2. The average time of the proposed method to detect a lesion image is about 150 ms, so that it runs sufficiently fast for real-time operation, since the additional computational costs for the lesion segmentation are negligible besides the real-time Gabor based feature extraction and classification. The proposed detector of malignant melanoma is designed and implemented for much of its framework using Microsoft Visual Studio 2016 and OpenCV Vision Library to realize real-time digital image processing and automatic detection. All tests and evaluations were carried out on a PC with an Intel(R) Core(TM) i7 CPU—2.60 GHz processor, 8GB RAM, running a Windows 10 Professional 64-bit operating system.

### Limitations

It is worth mentioning that the comparison of the current diagnostic approach with the two previous works in [28,29] seems to be only conditionally possible, since we used the benchmark PH2 dataset consisting of 200 dermoscopic images (40 malignant and 160 benign), and a 5-fold cross-validation (CV) technique was applied. On the other hand, the method presented in [28] relied on a different dataset (224 lesions in total, with 50% malignant), and a 4-fold CV technique was applied, while the method in [29] used a dataset consisting of 40 images, and applied an n-fold CV technique. Another limitation might be the lack of another independent dataset for validating the diagnostic model, since a dataset of 200 images might be too small to retain an initial set of images as an independent test set.

## 5. Conclusions

In this paper, a new CAD method for malignant melanoma detection has been proposed, using an optimized set of Gabor-based features and a fast MNN classifier with improved backpropagation based on the LM algorithm. On the publicly available PH2 dermoscopy imaging dataset, the proposed method has achieved an accuracy of 97.50%, sensitivity of 100%, specificity of 96.87%, for 5-fold cross-validation. The results provide evidence that the method is not only able to automatically discern malignant melanoma from benign nevi successfully, but also achieves consistent improvement over other state-of-the-art baselines. One of our future work is to develop a hybrid feature descriptor obtained by combining different color and texture features via a classifier fusion scheme, so we can further achieve a better approach for automatic lesion feature extraction. Another proposal for future work is to apply the proposed CAD method to larger dermoscopic image datasets to examine the consistency of its performance pattern.

## Figures and Tables

**Table 1:** Cross-classification: model’s prediction benign/malignant melanoma.

	Malignant	Benign
Test (+)	40	5
Test (−)	0	155

**Table 2:** Comparison of our methodology with other state-of-the-art baselines.

Method	SN (%)	SP (%)	AC (%)
Our Method	100	96.87	97.50
Bakheet [28]	98.21	96.43	97.32
Elgamal [29]	100	95.00	97.00
